# Infectious Disease: Tackling Innate Immunity

**DOI:** 10.1289/ehp.112-1247665

**Published:** 2004-11

**Authors:** Carol Potera

According to the 1999 World Health Organization report *Removing Obstacles to Healthy Development*, infectious diseases cause one-third of all human deaths worldwide. These diseases also cost the livestock industry billions of dollars yearly, according to figures from the U.S. Department of Agriculture National Center for Animal Health Surveillance. Infectious diseases are currently fought largely with vaccines (which generate so-called adaptive immunity) and antibiotics. But adaptive immunity can take months to acquire, and overuse of antibiotics may promote resistance in bacteria. If researchers with a Canadian project called Functional Pathogenomics of Mucosal Immunity (FPMI) can unlock the genetic mechanisms behind another branch of immunity—innate immunity—they may have the key to faster-acting, more effective medicines by harnessing the body’s rapid-response agents. Indeed, project scientists recently identified a highly promising peptide candidate for future immunotherapies.

FPMI is funded by Genome Canada, a nonprofit corporation dedicated to advancing genomics and proteomics to improve human and animal health. The three-year project involves groups at the University of Saskatchewan, the University of British Columbia, Simon Fraser University, and the Vancouver firm Inimex Pharmaceuticals.

“The unique strength of FMPI is the application of animal and human models of infection to study evolutionarily conserved host responses,” says microbiologist Vivek Kapur, co-director of the Biomedical Genomics Center at the University of Minnesota. Because the mechanisms of the innate immune system are not well understood, this comparative genomics approach to study host–pathogen interactions may lead to new immunotherapeutics to prevent infections, he adds.

Innate immunity appears highly conserved in evolution, suggesting that similar events occur in different species. Innate immunity is relatively nonspecific and acts rapidly to block pathogens at the point that they enter the body: the mucous membranes of the respiratory, digestive, and reproductive tracts. Agents produced by the innate immune system—such as cytokines, chemokines, and natural host defense peptides—act immediately in response to infection.

The researchers use microarrays to watch gene activity in humans and animals following exposure to six bacteria and three viruses associated with hospital-acquired infections, food poisoning, and livestock illnesses. “If we can show that the same genetic processes happen in cows, chickens, and humans, that gives us a great deal of confidence that we’re on the right path [to understanding the mechanism involved],” says project co-leader Lorne Babiuk, director of the Vaccine and Infectious Disease Organization at the University of Saskatchewan.

The data generated by the thousands of microarray experiments are processed by bioinformaticists headed by Fiona Brinkman, an assistant professor of molecular biology and biochemistry at Simon Fraser University. The team’s sophisticated software system, called ArrayPipe, “allows researchers from distant geographic regions to work together and view each others’ analyses,” says Brinkman. The software is available in an “open source” format that makes it very flexible and easy to customize. ArrayPipe can be downloaded for free at **http://www.pathogenomics.ca/arraypipe/**.

The genes related to innate immunity encode disease-fighting substances, which not only kill pathogens, but also produce inflammation. Although some inflammation is necessary to kill pathogens, it can escalate to undesirable conditions such as septic shock. One goal of the FPMI researchers is to find ways to induce desirable disease-fighting responses, yet quell undesirable ones related to inflammation.

A major breakthrough came when researchers in the laboratory of FPMI co-leader Bob Hancock, who is director of the Centre for Microbial Diseases and Immunity Research at the University of British Columbia, showed that the natural host defense peptide LL-37 cures infections as it suppresses inflammation. In a report published 15 March 2004 in *The Journal of Immunology*, Hancock and colleagues write that LL-37 up-regulates genes linked with the inflammation that kills microbes, but down-regulates those linked with the inflammation that promotes septic shock, suggesting that LL-37 serves as a watchdog to control inflammatory processes. “This . . . indicates that you can get the good aspects of innate immunity without the bad,” says Hancock.

Scientists at Inimex are designing new drug compounds based on LL-37. The new strategy will encourage the body’s innate immune system to attack foreign invaders, rather than bombard bacteria with antibiotics—an approach that increasingly leads to antibiotic-resistant strains. “It’s a new perspective that’s desperately needed to counteract antibiotic-resistant bacteria,” says Hancock.

## Figures and Tables

**Figure:**
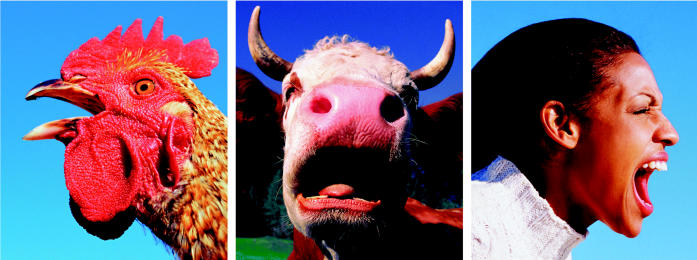
**Everybody’s got one.** The ability of innate immunity to block pathogens at the mucous membranes appears highly conserved across species.

